# Adriamycin-induced oxidative stress is prevented by mixed hydro-alcoholic extract of *Nigella sativa* and *Curcuma longa* in rat kidney

**Published:** 2016

**Authors:** Reza Mohebbati, Mohammad Naser Shafei, Mohammad Soukhtanloo, Noema Mohammadian Roshan, Abolfazl Khajavi Rad, Akbar Anaeigoudari, Sara Hosseinian, Sareh Karimi, Farimah Beheshti

**Affiliations:** 1 *Department of Physiology, School of Medicine, Mashhad University of Medical Sciences, Mashhad, Iran *; 2 *Neurocognitive Research Centerand department of Physiology, School of Medicine, Mashhad University of Medical Sciences, Mashhad, Iran*; 3 *Department of Biochemistry, School of Medicine, Mashhad University of Medical Sciences, Mashhad, Iran*; 4 *Department of Pathology, Qaem Hospital, Mashhad University of Medical Sciences, Mashhad, Iran *; 5 *Neurogenic Inflammation Research Center, Department of Physiology, School of Medicine, Mashhad University of Medical Sciences, Mashhad, Iran*; 6 *Department of Physiology, School of Medicine, Jiroft University of Medical Sciences, Jiroft, Iran*

**Keywords:** *Adriamycin*, *Nigella sativa*, *Curcuma longa*, *Oxidative stress*

## Abstract

**Objective:**

Inflammation and oxidative stress is considered to have a crucial role in induction of nephropathy. *Curcuma longa (C. longa) *and* Nigella sativa (N. sativa*) have anti-inflammatory and antioxidant effects. This study was designed to investigate the effect of mixed hydro-alcoholic extract of *N.sativa *and* C. longa* on the oxidative stress induced by Adriamycin (ADR) in rat kidney.

**Materials and Methods:**

The animals were divided into 6 groups: control (CO), ADR, Adriamycin+ Vitamin C (ADR+VIT C), *C. longa* extract+ Adriamycin (C.LE+ADR), *N. sativa* extract+ Adriamycin (N.SE+ADR) and *C. longa* extract+ *N. sativa* extract + Adriamycin (N.S+C.L+ADR). ADR (5mg/kg) was injected intravenously, whereas VITC (100mg/kg) and extract of *C. longa* (1000mg/kg) and *N. sativa* (200mg/kg) were administrated orally. Finally, the renal tissue, urine and blood samples were collected and submitted to measure of redox markers, osmolarity and renal index.

**Results:**

The renal content of total thiol and superoxide dismutase (SOD) activity significantly decreased and Malondialdehyde (MDA) concentration increased in Adriamycin group compared to control group. The renal content of total thiol and SOD activity significantly enhanced and MDA concentration reduced in treated-mixed extract of *C. longa* and *N. sativa* along with ADR group compared to ADR group. The mixed extract did not restore increased renal index percentage induced by ADR. There also was no significant difference in urine and serum osmolarity between the groups.

**Conclusion:**

hydro-alcoholic extracts of *N.sativa* and *C.longa* led to an improvement in ADR-induced oxidative stress and mixed administration of the extracts enhanced the aforementioned therapeutic effect.

## Introduction

Nephrotic syndrome (NS) as a glomerular pathological condition involves both children and adulthood. In adulthood NS is considered as the secondary disorder to diabetes mellitus, cancer and chronic inflammatory disease whereas in children is mainly primary or idiopathic (Cizmarikova et al., 2015 ▶). Among various contributing factors in the kidney diseases, inflammatory processes and oxidative stress play a crucial role in renal disorders including glomerulonephritis, nephropathy and acute renal injury (Rybi-Szuminska et al., 2014 ▶). In the nephropathy, the levels of reactive oxygen species (ROS) enhance and levels of antioxidant agents reduce (Medina-Navarro et al., 2014 ▶). Scientific findings indicated that nephrosis-induced damage in animal models is mediated by oxygen-free radicals (Nakakura et al., 2004 ▶). Adriamycin (ADR) (doxorubicin) as an anthracycline antibiotic was used in clinic due to its anticancer effects (Lee and Harris, 2011 ▶). In addition, the involvement of ADR and oxidative stress has been well documented. It has been suggested that ADR results in lipid peroxidation, generation of free radicals, consequently oxidative damage and nephrosis progression, hence, using ADR is limited in clinic (Quiles et al., 2002 ▶). Based on these findings it seems that using antioxidant agents could ameliorates ADR-induced oxidative stress in kidney. Nowadays herbal plants such as *Curcuma longa *(*C. longa*) (Venkatesan et al., 2000 ▶) and *Nigella sativa* (*N. sativa*) (Ahmad et al., 2013 ▶) are used for curing many diseases including renal disorders. *C. longa* possesses antioxidant and anti-inflammatory agents including curcumin, effective compound of *C. longa*, whose antioxidant power is equivalent to vitamins C, E and Beta-Carotene (Akram et al., 2001 ▶). It has been shown that curcumin inhibits formation of ROS and protects renal line cells against oxidative stress (Cohly et al., 1998 ▶). Curcumin also prevents renal lesions in streptozotocin-induced diabetic nephropathy (Babu and Srinivasan, 1997 ▶) and ADR-induced cardio-toxicity in rats (Venkatesan, 1998 ▶). Neuroprotective (Khazdair, 2015 ▶), renoprotective and antioxidant properties of *N. sativa* have also been documented. Thymoquinone, the effective compound of *N. sativa*, prevents diabetic nephropathy (Omran, 2014 ▶). It has been revealed that vitamin C and *N. sativa *oil both exert nephron-protective effects through lessening oxidative stress (Saleem et al., 2012 ▶). In spite of these reports, the effect of total extract of *C. longa *and *N. sativa *and using both extracts of these two herbal plants on renal disease has not been reported, therefore, the aim of the present study was evaluation of antioxidant effect of hydroalcoholic extract of *C. longa *and *N. sativa* on ADR- induced oxidative stress in rats. 

## Materials and Methods

**Plant material and preparation of the extract**

*C. longa* rhizomes and *N. sativa* seeds were purchased from a local herbal shop in Mashhad, Khorasan province, Iran and identified by botanists in the herbarium of Ferdowsi University of Mashhad and the specimen number of the plant is 293-0303-1.

*C. longa* rhizomes (100 g) and *N. Sativa* seeds (100g) were cleaned, dried, ground, weighed, and homogenized in 70% ethanol at a ratio of 1:10 of plant to ethanol and left to soak for 3 days at 37°C with occasional shaking and stirring. The mixture was then filtered and the resulting liquid was concentrated under reduced pressure at 45°C in an EYELA rotary evaporator (7%, w/w). The concentrated extract was then kept in the incubator at 45°C for 3 days to evaporate the ethanol residue yielding the crude extract (Salama et al., 2013 ▶). The extracts were then dissolved in 96% ethanol (0.5%, w/w) before being orally administrated to animals.

**Chemicals and drugs**

The used chemicals for biochemical assessments were purchased from Merck Company. ADR was obtained from EBO pharma, Iran.

**Animals and treatment**

Eighty Male Wistar rats (220 - 250 g, 10 weeks old) were purchased from animal house of Mashhad University of Medical Sciences, Mashhad, Iran. The animals were housed in a room with standard temperature (22±2ºC) and 12 h light/dark cycle. The rats were allowed to food and water freely. All experiments were performed under the license from the Animal Experimentation Ethics Committee of Mashhad University of Medical Sciences.

Animals were randomly divided ino six groups including: Control (CO), Adriamycin (ADR), Adriamycin plus Vitamin C (ADR+VIT C), *C. longa *extract plus Adriamycin (C.LE+ADR), *N. sativa *extract plus Adriamycin (N.SE+ADR) and *C. longa *extract plus *N. sativa *extract plus Adriamycin (N.S.+C.L+ADR). ADR (5mg/kg) (Zima, 1998 ▶) was injected intravenously, whereas VIT C (100mg/kg) (Greggi, 2000) and extract of *C. longa *(1000mg/kg) (Khorsandi, 2008 ▶) and *N. sativa* (200mg/kg) (dose of N.S optimized by pilot study) were administrated orally. 

**Preparation of rat renal tissue**

At the end of the treatment period, the animals were euthanized by decapitation with guillotine. The renal tissue was rapidly removed and stored at -80 ° C after washing.

**Malondialdeyde (MDA) and thiol assessment**

The kidney samples were homogenized with ice-cold KCl (150 mM) for the determination of MDA and thiol levels.

**Determination of MDA concentration**

MDA level is as an index of lipid peroxidation. MDA reacts with thiobarbituric acid (TBA) as a TBA reactive substance (TBARS) and produces a red complex. Briefly, 1 mL of homogenates was added to 2 mL of a complex solution containing TBA/trichloroacetic acid (TCA) /hydrochloric acid (HCL) and it was then boiled in a water bath for 40 minutes. After reaching the room temperature, the solution was centrifuged at 1000 g for 10 minutes. The absorbance was read at 535 nm (Janero, 1990 ▶). The MDA concentration was calculated according to the following equation.

MDA concentration (M) = Absorbance / (1.56 × 105 cm-1 M-1)

The MDA levels results are expressed per gram of tissue.

**Determination of thiol concentration**

DTNB (2, 2'-dinitro-5, 5'-dithiodibenzoic acid) reagent, which reacts with the SH group, was used to determine the total thiol groups. The produced yellow complex has a peak absorbance at 412 nm. Briefly, 50 μL of tissue homogenates was added to 1 ml Tris-EDTA) ethylenediaminetetraacetic acid) buffer (pH = 8.6) and the absorbance was read at 412 nm against Tris-EDTA buffer alone (A1). Then, 20 μL of 10 mM solution of DTNB was mixed with the solution and it was stored in room temperature for 15 minutes and the absorbance was read again (A2). The absorbance of DTNB reagent was also read as blank (B) (Sharma et al., 2006 ▶). The thiol levels were determined by a spectrophotometric method based on the use of Ellman’s reagent and the results are expressed as per gram of tissue. 

Total thiol concentration (mM) = (A2 – A1 – B) × 1.07) / 0.05 × 14, 150.

**Determination of superoxide dismutase (SOD) activity**

SOD activity was measured by the procedure of Madesh and Balasubramanian. A colorimetric assay involving generation of superoxide by pyrogallol auto-oxidation and the inhibition of superoxide-dependent reduction of the tetrazolium dye, MTT (3-(4, 5-dimethylthiazol-2-yl) 2, 5-diphenyltetrazolium bromide) to its Formosan by SOD was measured at 570 nm. One unit of SOD activity was defined as the amount of enzyme causing 50% inhibition in the MTT reduction rate (Madesh and Balasubramanian, 1998 ▶). 

**Determination of renal index percentage and osmolarity**

Renal index was calculated according to the following formula: 

Renal index= kidney weight/body weight

Also, the osmolarity was measured by Osmomat 030 (gonatec CO, Iran).

**Statistical analysis**

All data were expressed as means ± SEM. Normality test (Kolmogorov–Smirnov) was done, too. Different groups were compared by one way ANOVA followed by tukey's Post Hoc comparison test. Differences were considered statistically significant when p<0.05.

## Results

**Figure 1:**
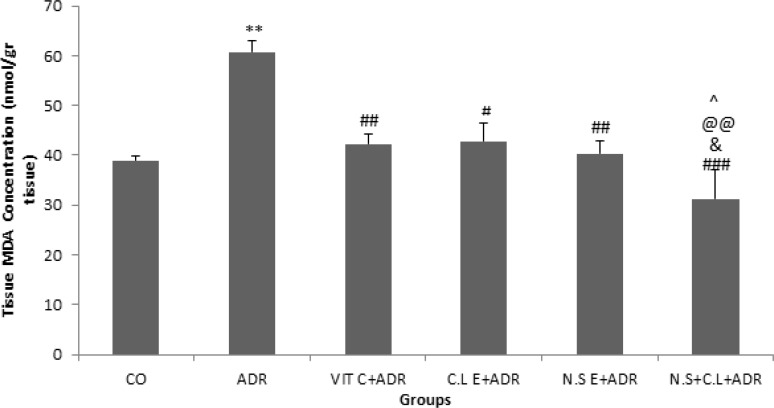
Comparison of the MDA concentrations in renal tissue of experimental groups. Data are presented as Mean ± SEM (n= 8 in each group). **p<0.01 compared to control group. #p<0.05, ##p<0.01 and ###p< 0.001 compared to ADR group. &p<0.05 compared to N.S E+ ADR group. @@p<0.01 compared to C.L E + ADR group. ^p<0.05 compared to VIT C + ADR group

The results of current study indicated that the level of MDA was higher in ADR group compared to CO group (p<0.01). In addition, the concentration of MDA in groups ADR + VIT C, ADR+ C.L E, ADR + N.S E and ADR + N.S + C.L significantly decreased in comparison with ADR group (p<0.05, p<0.01 and p<0.001). The level of MDA reduced significantly in ADR + N.S + C.L group with respect to ADR + VIT C, ADR+ C.L E and ADR + N.S E groups (p<0.05) (Figure 1).

The results revealed that the level of total thiol content was lower in ADR group compared to CO group (p<0.01). The concentration of total thiol content in groups ADR + VIT C, ADR+ C.L E, ADR + N.S E and ADR + N.S + C.L significantly increased in comparison with ADR group (p<0.01 and p<0.001). The level of total thiol content enhanced significantly in ADR + N.S + C.L group with respect to ADR + VIT C and ADR + N.S E groups (p<0.05).

**Figure 2:**
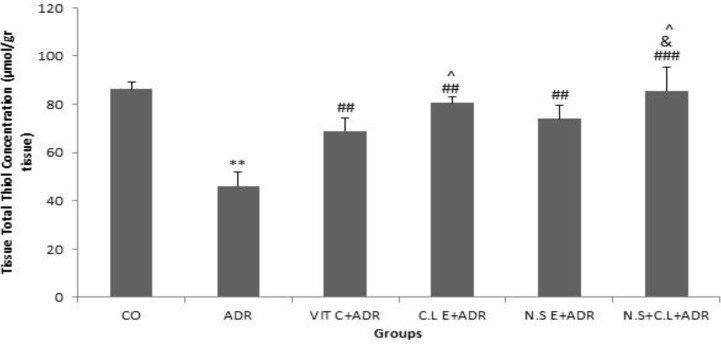
Comparison of the total thiol concentration in renal tissue of experimental groups. Data are presented as Mean ± SEM (n= 8 in each group). **p<0.01 compared to control group. ##p<0.01 and ###p< 0.001 compared to ADR group. &p<0.05 compared to N.S E+ ADR group. ^p<0.05 compared to VIT C + ADR group.

Additionally, the level of total thiol content was higher in C.L E + ADR compared to VIT C + ADR group (p<0.05) (Figure 2).

**Figure 3:**
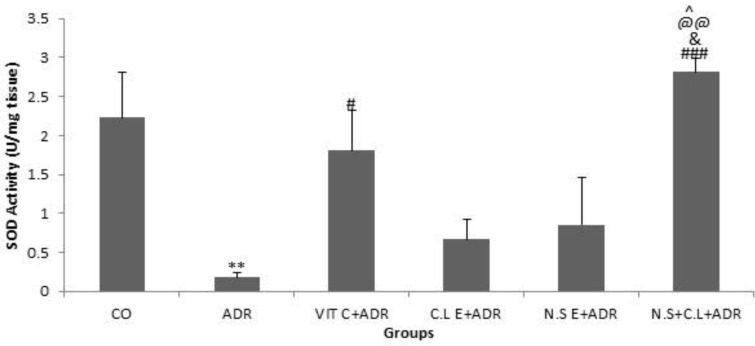
Comparison of the SOD activity in renal tissue of six groups. Data are presented as Mean ± SEM (n= 8 in each group). **P<0.01 compared with control group. #P<0.05 and ###P< 0.001 compared with ADR group. &P<0.05 compared to N.S E+ ADR group. @@P<0.01 compared to C.L E + ADR group. ^P<0.05 compared to VIT C + ADR group

The results showed that the SOD activity was lower in ADR group compared to CO group (p<0.01). The SOD activity in groups ADR + VIT C and N.S + C.L + ADR significantly increased in comparison with ADR group (p<0.05 and p<0.001). The SOD activity enhanced significantly in N.S + C.L + ADR group with respect to ADR + VIT C, ADR + N.S E and C.L + ADR groups (p<0.05 and p<0.01) (Figure 3).

**Figure 4:**
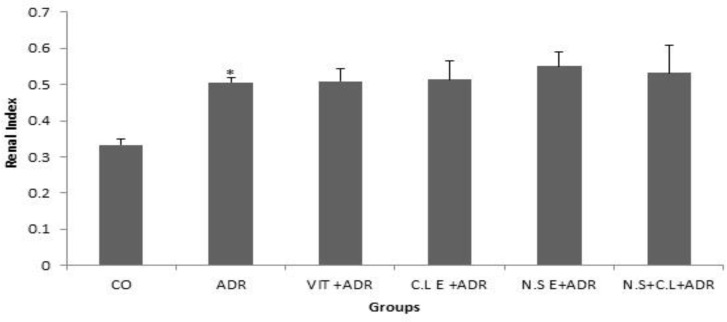
Comparison of renal index percentage in six groups. Data are presented as Mean ± SEM (n= 8 in each group). *p<0.05 compared with control group

Also the results showed that the renal index percentage was higher in ADR group compared to CO group (p<0.05), while the other groups did not see the significant difference compared to ADR group (Figure 4).

**Figure 5:**
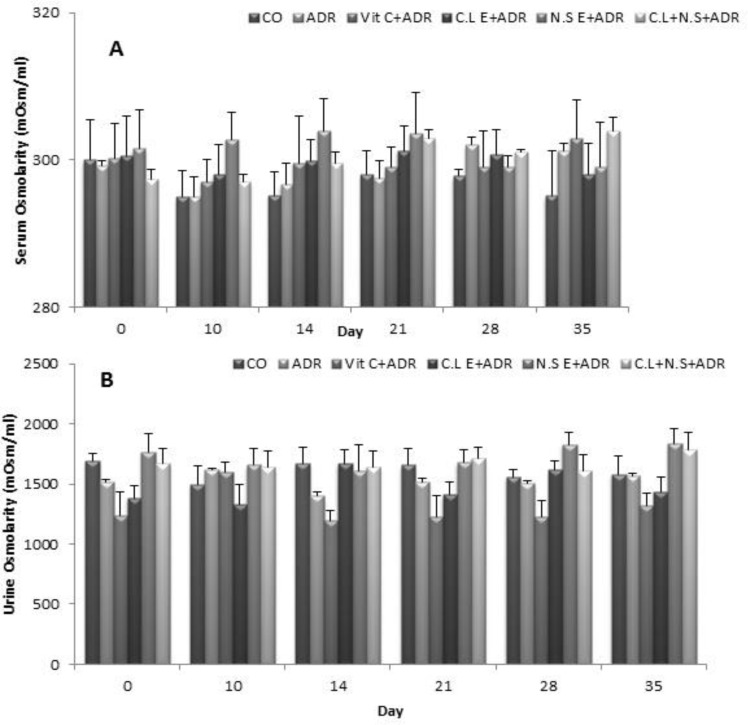
Comparison of urine and serum osmolarity between the six groups in different days. Data are presented as Mean ± SEM (n= 8 in each group). There was no significant difference in urine and serum osmollarity between groups

In addition, we did not observe any significant difference in the urine and serum osmolarity between the six groups in different days (Figure 5A and 5B).

## Discussion

The results of the current study showed that injection of ADR leads to renal tissues oxidative damage. Accordingly, tissue concentration of MDA increased and the total thiol content and SOD activity reduced in the renal tissue of ADR-treated animals in comparison with the control group. The renal tissues oxidative damage of ADR has also been shown previously. Sarhan et al have reported that intravenous administration of ADR increases the level of MDA and decreases the total thiol content in the renal tissue (Sarhan et al., 2014 ▶). In addition, administration of ADR results in decreasing the level of antioxidant enzymes such as catalase, glutathione peroxidase and SOD and enhancing MDA concentration in renal tissues of mice (Jadhav et al., 2013 ▶). All these reports confirm the results of the present study.

The present work indicated that the administration of *N. sativa* and *C. longa*, separately, decreased tissue MDA level but increased total thiol and SOD activity in the kidney tissue. Some evidence indicates that C. longa and N. sativa have antioxidant, anti-inflammatory and cell membrane stabilizing effects (Ahmad et al., 2013 ▶). The antioxidant effects of C. longa are attributed to the presence of curcumin. Curcumin is able to inhibit lipid peroxidation in the renal microtomes and mitochondria in a dose-dependent manner (Venkatesan et al., 2000 ▶). It has also been reported that the administration of curcumin can enhance the content of glutathione, glutathione peroxidase activity in renal tissues in ADR-treated rats (Sharma, 1976 ▶). The antioxidant effects of N. sativa are mostly related to thymoquionone. Thymoquoinone has been shown to have beneficial effects on oxidative stress, hyper- proliferative response and renal carcinogenesis in wistar rats (Khan and Sultana, 2005 ▶). Intraperitoneal injection of thymoquoinone showed to have protective effects on lipid peroxidation and to improve oxidative stress in rats (Hosseinzadeh et al., 2007 ▶). 

However, in the present investigation administration of the mixed extract of *N. sativa* and *C. longa* imply better antioxidant effects on the kidney tissue than either extracts. The mixed extract was able to significantly reduce renal tissue MDA concentration and increase antioxidant markers including SOD and total thiol concentration in the rat kidney compared with all other treated animals. It seems that the mix administration of both *N. sativa *and* C. longa* extract potentiate antioxidant effects of either extracts. The mechanism potentiating antioxidant effect of the mixed extracts is unknown and needs further studies. However, it may be attributed to the synergic action of various components in both extracts including thymoquoinone and Curcumin. In addition, presence of flavonoids as common antioxidant components in C. longa and N. sativa may be involved in improving their effects on ADR-induced renal oxidative damage in the current study (Ahmad et al., 2013 ▶ and Akram et al., 2001 ▶).

Administration of the extracts separately and mixed did not affect ADR-increased renal index. It is believed that the current protocol was not able to affect ADR-induced renal edema in the extract-treated animals compared with the ADR group. However, it needs further investigations. 

Meanwhile, there was no significant difference in urine and serum osmolarity between all groups. Our results probably indicate that administration of ADR and extracts according to this investigation has no significant action on the kidney tubular function. Again, it needs more studies to clarify the exact effect of ADR and extracts on the renal tubular processes.

The present study showed that the administration of hydro-alcoholic extracts of *N. sativa *and* C. longa*, separately, were able to decrease the oxidative marker MDA but increase the antioxidants including SOD and total thiol concentrations in the rat ADR-induced kidney damage. However, co-administration of both extracts of *C. longa *and* N. sativa* potentiate these antioxidant effects in the rat kidney. More investigations need to clarify the exact mechanisms.
